# Effects of Sweating and Drying Processes on Chemical Components, Antioxidant Activity, and Anti-Acute Liver Injury Mechanisms of *Eucommia ulmoides* Based on the Spectrum–Effect Relationship

**DOI:** 10.3390/ijms26178686

**Published:** 2025-09-05

**Authors:** Peiyao Shi, Meng Zhang, Changxin Qian, Liangshi Lin, Qi Liu, Juan Xue, Shanshan Liang

**Affiliations:** 1 College of Pharmacy, Guizhou University of Traditional Chinese Medicine, Guiyang 550025, China; 18383256463@163.com (P.S.); 15599106414@139.com (M.Z.); 17685301617@163.com (C.Q.); 17878320921@163.com (L.L.); liuqik2025@163.com (Q.L.); xuejuan062@gzy.edu.cn (J.X.); 2 S Provincial Inheritance Base of Traditional Chinese Medicine Processing Under National Administration of Traditional Chinese Medicine, Guizhou University of Traditional Chinese Medicine, Guiyang 550025, China

**Keywords:** *Eucommia ulmoides* Oliv., sweating–drying processes, antioxidant activity, acute liver injury (ALI)

## Abstract

To investigate how sweating–drying processing affects the components, antioxidant activity, and hepatoprotective mechanisms of *Eucommia ulmoides* (EUB) against acute liver injury (ALI), this study constructed a “processing–active components–ALI targets” network. Eight processed EUB samples were analyzed using HPLC fingerprinting, multi-assay antioxidant tests (DPPH/ABTS·+/pyrogallol), network pharmacology, and molecular docking. Sweating–drying significantly altered EUB’s chemical profile, with HPLC fingerprint similarities ranging from 0.715 to 1.000, the lowest being for FG4 (40 °C dried after sweating) and FD (freeze-dried after sweating). Key components (chlorogenic acid (CA), pinoresinol diglucoside (PDG), aucubin (AU), geniposidic acid (GPA)) varied: XS (sun-dried) had the highest CA/PDG, while FG4 showed increased AU/GPA. FY (shade-dried after sweating) exhibited the strongest free radical scavenging (DPPH/ABTS·+/pyrogallol IC_50_ = 0.828, 0.134, 14.200 mg/mL), which correlated with CA/PDG/liriodendrin (PD) synergy. Network pharmacology identified 205 EUB-ALI intersection targets (core: TNF, PTGS2, GAPDH) and the AGE-RAGE pathway; molecular docking confirmed strong CA/PDG binding to GAPDH/PTGS2. This study clarifies how processing regulates EUB’s components and their links to antioxidant and hepatoprotective effects, providing scientific support for EUB’s clinical application against ALI.

## 1. Introduction

The quality consistency of Chinese herbal decoction pieces often relies on the standardization of processing techniques [1], among which “sweating” and “drying” are key steps to regulate the efficacy of Chinese herbs [2]. In traditional Chinese medicine (TCM) theory, “sweating” is regarded as an important processing technique. It promotes biochemical reactions within medicinal materials under appropriate temperature and humidity conditions to achieve component transformation, thereby enhancing therapeutic efficacy [3]. This theory has been validated by long-term clinical practice: In TCM, sweating is believed to alleviate the harshness of medicinal materials, facilitate the conversion of inactive components to active ones, and strengthen synergistic interactions among multiple components, rendering the efficacy more targeted and stable [4,5]. However, the scientific basis for how “sweating” regulates the chemical components and therapeutic effects of medicinal materials remains unclear, which limits the standardization of processing techniques and the rational clinical application of processed products.

*Eucommia ulmoides* Oliv. (EUB), a traditional Chinese herb for nourishing the liver and kidney, contains active components such as pinoresinol diglucoside, aucubin, and liriodendrin, which are susceptible to processing techniques. For instance, aucubin content decreases after sweating [6], while research conclusions on post-sweating changes in pinoresinol diglucoside content remain inconsistent [7,8].

As a core technique for holistic characterization of Chinese herb components [9], HPLC fingerprinting can directly reflect the impact of “sweating–drying” processes on the chemical components of EUB.

Preliminary studies in this research established HPLC fingerprints and found that the similarity between fingerprints of EUB samples FG4 (the samples were dried at 40 °C for 5 days after 10 days of sweating) and FD (the samples were freeze-dried for 3 days after 10 days of sweating) was only 0.715, suggesting that drying methods significantly affect chemical composition.

However, the correlation between these chemical changes and pharmacological activity has not been clarified through systematic spectrum–effect relationship studies.

Spectrum–effect relationship refers to analyzing the correlation between traditional Chinese medicine fingerprints (holistic characterization of chemical components) and pharmacodynamic activity, so as to elucidate the synergistic mechanism of key pharmacodynamic components.

Acute liver injury (ALI) is a severe clinical disease characterized by the rapid deterioration of hepatocyte function [10]. During liver injury, oxidative stress is a key pathological link in the occurrence and progression of ALI. Excessive free radicals (e.g., DPPH, ABTS·+) in hepatocytes can induce lipid peroxidation and DNA damage, thereby activating Caspase-3-mediated hepatocyte apoptosis [11].

For example, in acetaminophen (APAP)-induced ALI, the accumulation of reactive oxygen species (ROS) directly leads to mitochondrial dysfunction and hepatocyte death [12]. Moreover, oxidative stress can activate inflammatory pathways, further exacerbating liver injury [13].

In modern pharmacological studies, EUB has demonstrated notable antioxidant and hepatoprotective potential. Its extract can significantly reduce serum Alanine Transaminase (ALT) and Aspartate Transaminase (AST) levels in mice with carbon tetrachloride (CCl_4_)-induced liver injury and inhibit lipid peroxidation in liver tissue by enhancing the activity of Superoxide Dismutase (SOD) and Glutathione (GSH) while reducing malondialdehyde (MDA) content [14]. Furthermore, in chronic liver injury models, EUB extract can significantly improve liver function and liver tissue morphology by downregulating pro-inflammatory factors (e.g., IL-1β) and upregulating antioxidant enzyme activity [15]. Flavonoids in EUB (e.g., quercetin) act as scavengers of most oxygen free radicals, strengthening the antioxidant defense system by activating the Nrf2 pathway [16]; they also enhance SOD activity, showing significant efficacy in treating conditions such as coronary heart disease and hypertension [17]. Total flavonoids in EUB can significantly scavenge ABTS·+ and DPPH free radicals [18].

Moreover, phenolic acids, the most active antioxidants in plants, decrease the secrease of inflammatory factors by inhibiting the NF-κB pathway [19]. There are 41 reported phenolic acid compounds in EUB, among which chlorogenic acid is the primary antioxidant active component [20,21]. Lignans such as pinoresinol diglucoside have also been verified to possess strong antioxidant capacity, significantly scavenging ABTS·+ and DPPH free radicals [22].

Although existing studies have confirmed the hepatoprotective and antioxidant effects of EUB, they mostly focus on monomeric components. The mechanism by which “sweating–drying” processes influence acute liver injury (ALI)-related signaling pathways (e.g., AGE-RAGE, MAPK, Nrf2) via regulating the synergistic action of multiple components remains unclear. More importantly, the TCM theory that “sweating enhances the efficacy of EUB” lacks scientific validation, restricting the integration of traditional experience and modern science in the field of Chinese medicinal processing.

This study aims to fill these gaps. By comparing eight processing techniques of EUB decoction pieces and integrating HPLC fingerprint similarity analysis, multi-index antioxidant assays (DPPH/ABTS·+/pyrogallol), and network pharmacology analysis of acute liver injury, we will construct a multi-level regulatory network of “processing technique–active component–ALI target”. The research objectives include the following: (1) clarifying the regulatory rules of sweating and drying processes on the chemical components of EUB; (2) revealing the spectrum–effect relationship between processing-induced component changes and antioxidant/hepatoprotective activities; and, (3) based on network pharmacology and molecular docking, elucidating the material basis and key pathways of processed EUB against acute liver injury.

By achieving these objectives, this study intends to provide experimental evidence for the material basis of the TCM theory of “sweating–enhanced efficacy”, elucidate the scientific connotation of “sweating–enhanced efficacy” from the perspective of multi-component synergy, and offer a scientific basis for the standardization of EUB processing techniques and their precise clinical application in anti-acute liver injury. This work bridges traditional Chinese medicinal processing experience with modern pharmacological research, promoting the inheritance and innovation of Chinese medicinal processing theory.

## 2. Results

### 2.1. Determination of Sample Moisture

The *Chinese Pharmacopoeia (2020 Edition)* specifies that the moisture content of this product shall not exceed 13%. All samples conformed to this standard, with detailed results shown in Table 1. Under the same drying method, the moisture content of sweating-treated samples was lower than that of fresh samples. This finding indicates that the “sweating” process promotes more thorough drying of EUB, consistent with previous literature reports.

### 2.2. Generation and Similarity Analysis of Fingerprint Profiles

**Figure 1:**
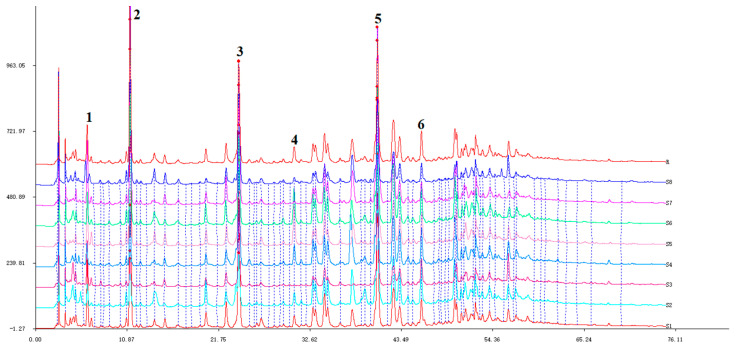
Comparative HPLC fingerprints of EUB processed using different methods. (X-axis: retention time (min); Y-axis: peak area. Peak.1: Aucubin, Peak.2: Geniposidic acid, Peak.3: Chlorogenic acid, Peak.4: Geniposide, Peak.5: Pinoresinol diglucoside, Peak.6: Liriodendrin).

**Figure 2:**
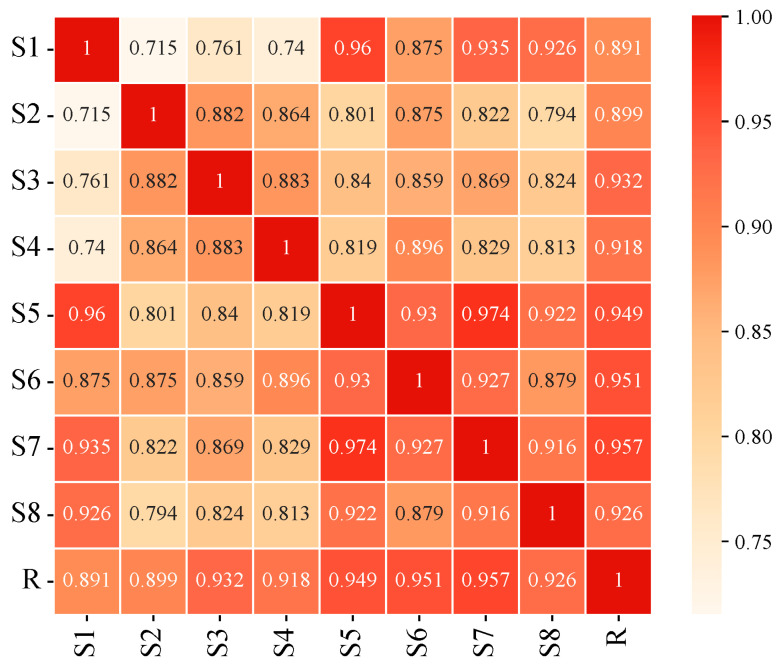
Comparative similarity of fingerprints among different processing methods (the color gradient indicates the similarity value; the darker the color, the higher the similarity).

The chromatographic data of various EUB samples were imported into the “Software for Similarity Evaluation of TCM Chromatographic Fingerprints” for chromatographic peak matching with a time window of 0.10, and the overlaid fingerprints are shown in Figure 1. The consistency of chromatographic peak similarity was analyzed by the median method, with results presented in Figure 2.

The similarity of common peaks in samples relative to the reference fingerprint (R) (with S1 as the reference peak—S1 showed complete and well-separated chromatographic peaks, covering the major characteristic peaks of EUB) ranged from 0.715 to 1.000, indicating differences in EUB processed by different methods. Among all samples, S5 and S7 showed the highest similarity (0.974).

For samples after sweating, the similarity among different drying methods ranged from 0.715 to 0.883, with S3 and S4 being the most similar (0.883); S1 and S2 exhibited the lowest similarity (0.715), with S1 differing significantly from the other three. For fresh samples, the similarity among different drying methods ranged from 0.879 to 0.974, with S5 and S7 being the most similar (0.974), and S6 and S8 exhibiting the lowest similarity (0.879).

The similarities of samples with the same drying method before and after sweating were 0.96, 0.875, 0.869, and 0.813, respectively. The high similarity before and after sweating indicates that the “sweating” process can well preserve the original components in EUB.

### 2.3. Determination of the Contents of Six Components in Samples

The contents of six pharmacodynamic components in EUB samples processed by different methods were determined according to the procedure under “4.4.3” (Table 2).

Among them, S7 showed the highest contents of AU, GPA, CA, and PDG; S5 had the highest contents of GP and PD. Among samples after sweating, S1 contained the highest total active components; S2 had the highest PDG content among post-sweating samples.

In fresh samples, S7 had the highest total active components (the sum of the contents of the six pharmacodynamic components quantified) and PDG content, indicating that sun-drying fresh samples is more conducive to the accumulation of active components. It can also be observed that different drying methods result in differences in the contents of pharmacodynamic components.

For samples before and after sweating, after being uniformly dried at 40 °C, the contents of AU and GPA increased in post-sweating samples, while the other four components decreased. In freeze-dried samples, all components decreased after sweating; in sun-dried samples, except for PD (content increased after sweating), the other components also decreased. In shade-dried samples, except for GPA and CA (contents decreased after sweating), the other four components increased. The results indicated that under the same drying method, sweating treatment exerts different effects on pharmacodynamic components.

### 2.4. Results of Antioxidant Activity Determination

The half-maximal inhibitory concentration (IC_50_) refers to the concentration of a sample solution that scavenges half of the free radicals; a smaller IC_50_ indicates a better free radical scavenging effect of the test substance and stronger antioxidant capacity. The free radical scavenging rates were calculated using formulas, and the IC_50_ values of the EUB samples obtained from the analysis are shown in Table 3.

S4 exhibited the strongest scavenging rates against DPPH, ABTS·+, and pyrogallol free radicals, suggesting that shade-drying after sweating can significantly enhance the scavenging capacity of EUB against these three free radicals.

The comparison of results before and after sweating under the same drying method showed that, via the DPPH assay, all samples except the freeze-dried ones exhibited stronger antioxidant capacity after sweating. The ABTS·+ assay showed the opposite trend: Only shade-dried samples had increased activity after sweating, while non-sweated samples performed better under other drying methods. The results of the pyrogallol assay differed from the previous ones: Freeze-dried and shade-dried samples showed stronger antioxidant capacity after sweating, whereas the opposite was true for oven-dried and sun-dried samples.

### 2.5. Grey Relational Analysis (GRA)

**Figure 3:**
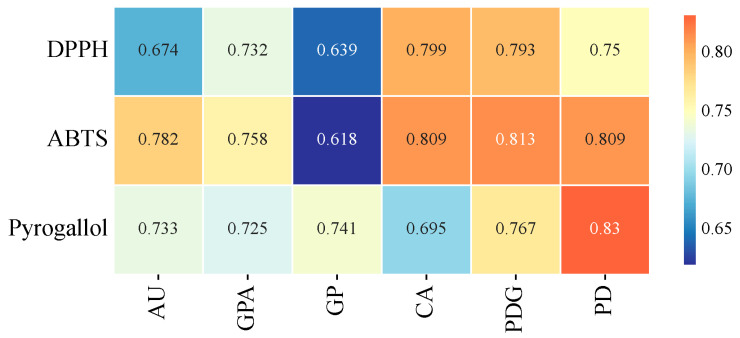
The results of Grey correlation analysis (the color gradient indicates the correlation coefficient “r”; the darker the color, the higher the “r”).

Grey relational analysis of the contents of each component and IC_50_ (Figure 3) showed that all relational degrees (r) were greater than 0.618. Among them, the contents of CA and PDG had relatively high relational degrees with the capacity to scavenge DPPH free radicals (r = 0.799, 0.793), indicating that the antioxidant effects of these two components play a major role in scavenging DPPH free radicals. Similarly, the contents of CA, PDG, and PD had high relational degrees with the capacity to scavenge ABTS·+ free radicals (r = 0.809, 0.813, 0.809), suggesting that the antioxidant effects of these components are significant in scavenging ABTS·+ free radicals. For pyrogallol free radicals, it mainly showed a high relational degree with PD (r = 0.830), indicating that PD is the main selective component exerting antioxidant effects in scavenging pyrogallol free radicals.

### 2.6. Results of Network Pharmacology

#### 2.6.1. Prediction of Targets for Acute Liver Injury in EUB Components

**Figure 4:**
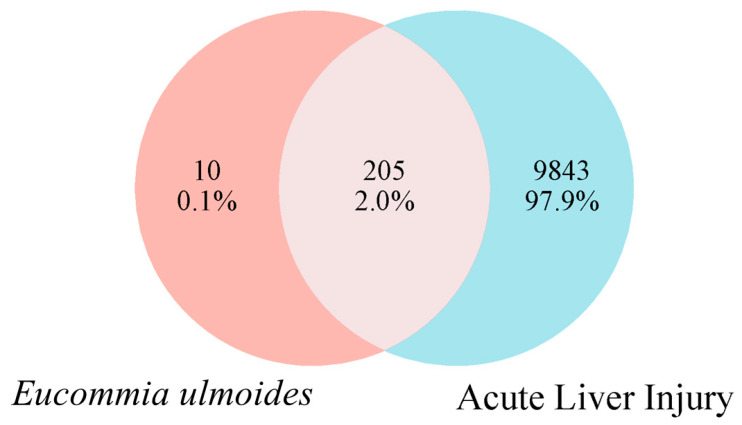
Intersectional targets between components of EUB and acute liver injury.

After literature review and database correction, we selected 33 components (including PDG, AU, GPA, GP, etc.) as representative components of EUB for network pharmacology research and sorted them by degree value (Table 4). Target proteins corresponding to the 33 active components were identified via the UniProt database, and a total of 215 target proteins with a probability value >1 were screened out. Using “Acute liver injury” as the keyword, 10,048 target genes related to acute liver injury were retrieved from the OMIM and GeneCards databases. Through Venn analysis to obtain the intersection, 205 common targets were identified (Figure 4).

#### 2.6.2. Construction of Protein–Protein Interaction (PPI) Network and Screening of Network Cores

**Figure 5:**
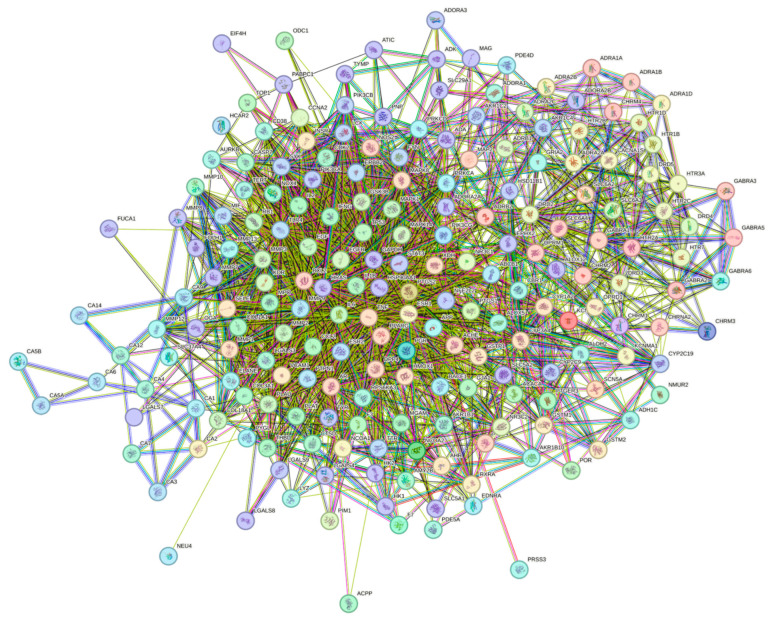
PPI network interaction of EUB and acute liver injury.

**Figure 6:**
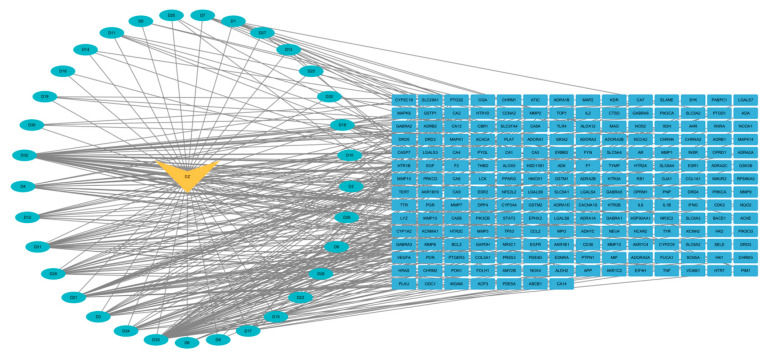
Drug–component–target network diagram (the orange nodes represent EUB, the green nodes represent the active components of EUB, and the blue nodes represent the targets of EUB).

**Figure 7:**
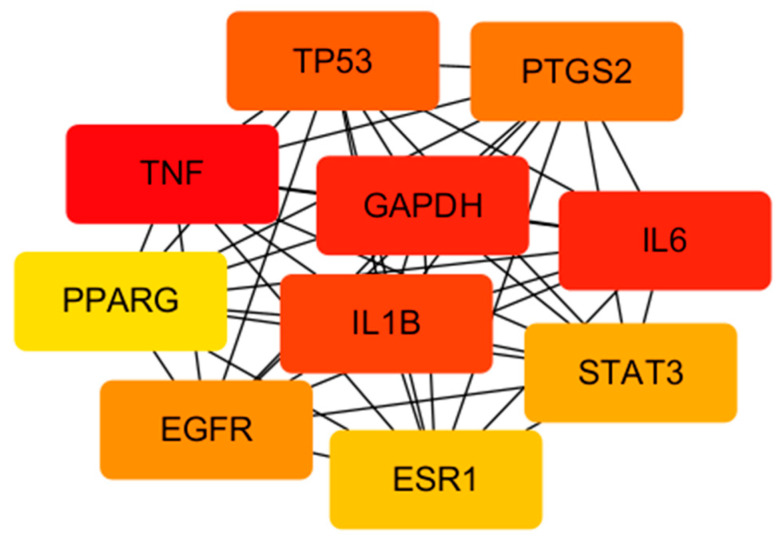
The CytoNCA plugin screens out the top 10 core target proteins through two median filters (the depth of color represents the association strength; the deeper the red, the stronger the association, and yellow are relatively weaker).

The 205 intersection targets between EUB components and acute liver injury were imported into the STRING database, yielding a PPI network. Core targets were screened via the Tools function of Cytoscape software and visualized (Figure 5). After constructing the “Network.txt” and “Tape.txt” files, they were imported into Cytoscape software. Using the MCC algorithm in the CytoHubba and CytoNCA plugins, the degree values were calculated to construct the “drug–active component–target” network diagram (Figure 6). The screened core targets included TNF, IL6, GAPDH, IL1B, TP53, PTGS2, EGFR, STAT3, ESR1, and PPARG (with degree > 140), which exhibit high connectivity and betweenness centrality in the network (Table 5, Figure 7). With a degree value ≥6 as the criterion, the screened active components included geniposide, caffeic acid, quercetin, chlorogenic acid, rutin, kaempferol, β-sitosterol, syringetin, (−)-Tabernemontanine, eudesmin, dehydrodiconiferyl alcohol, pinoresinol, quinidine, aucubin, pinoresinol diglucoside, protocatechuic acid, and octyl p-methoxycinnamate (Table 6).

#### 2.6.3. Pathway Analysis Results

**Figure 8:**
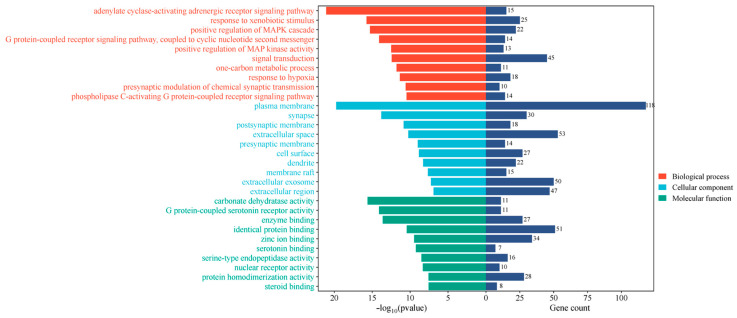
Results of gene ontology (GO) analysis.

The 205 obtained intersection targets were imported into the DAVID database, and “OFFICIAL GENE SYMBOL” was selected for GO and KEGG analyses. GO analysis involved 618 biological processes (BPs), 90 cellular components (CCs), and 154 molecular functions (MFs). Among them, BPs mainly included adenylate cyclase–activating adrenergic receptor signaling pathway, response to exogenous stimuli, positive regulation of MAPK cascade, etc.; CCs mainly involved plasma membrane, synapse, postsynaptic membrane, etc.; MFs mainly included carbonic anhydrase activity, G protein coupling, 5-hydroxytryptamine receptor activity, enzyme binding, etc. (Figure 8).

**Figure 9:**
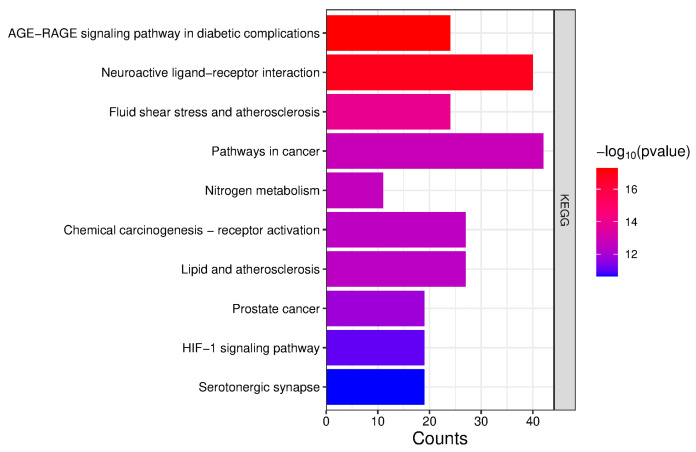
Results of KEGG analysis.

KEGG (Kyoto Encyclopedia of Genes and Genomes) analysis involved 160 signaling pathways, mainly including the AGE–RAGE signaling pathway in diabetic complications, neuroactive ligand–receptor interaction pathway, fluid shear stress and atherosclerosis pathway, etc. (Figure 9).

### 2.7. Results of Molecular Docking

**Figure 10:**
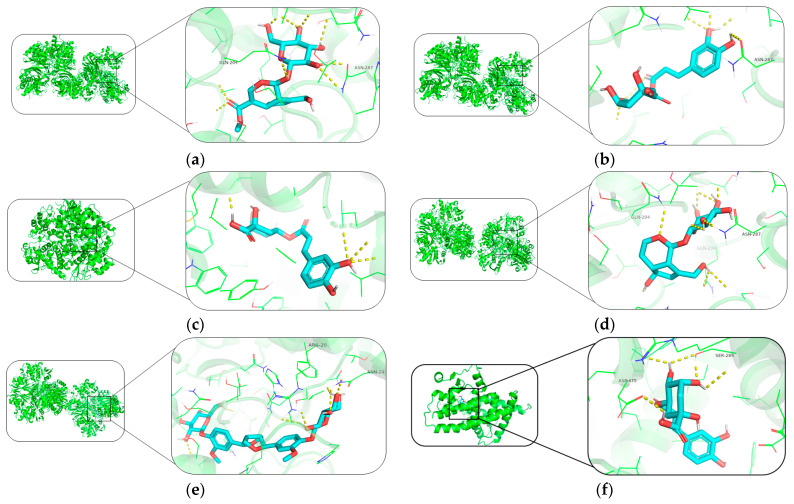
Visualization of the results of the molecular docking (the green represents receptor proteins, blue represents compounds and red represents key active sites, chemical bonds, or interaction regions). (**a**) Geniposide–GAPDH, (**b**) chlorogenic acid–GAPDH, (**c**) chlorogenic acid–PTGS2, (**d**) aucubin–GAPDH, (**e**) pinoresinol diglucoside–GAPDH, and (**f**) pinoresinol diglucoside–PTGS2, respectively.

Ten target proteins with the top 10 degree values were selected: TNF (PDB ID: 5uui), IL6 (PDB ID: 1alu), GAPDH (PDB ID: 6ynd), IL1B (PDB ID: 8rys), TP53 (PDB ID: 3D06), PTGS2 (PDB ID: 5F19), EGFR (PDB ID: 8A27), STAT3 (PDB ID: 6NJS), ESR1 (PDB ID: 8BZW), and PPARG (PDB ID: 8FKC). These were subjected to molecular docking with active components including geniposide, caffeic acid, quercetin, chlorogenic acid, rutin, kaempferol, β-sitosterol, syringetin, (−)-Tabernemontanine, (+)-Eudesmin, dehydrodiconiferyl alcohol, pinoresinol, quinidine, aucubin, pinoresinol diglucoside, protocatechuic acid, and octyl p-methoxycinnamate. Molecular docking was performed using AMDock software, and the results are shown in Table 7. Among them, geniposide showed good docking effect with GAPDH; chlorogenic acid exhibited good docking effects with GAPDH and PTGS2; aucubin showed good docking effect with GAPDH; pinoresinol diglucoside had good docking effects with GAPDH and PTGS2 (Figure 10).

## 3. Discussion

### 3.1. Regulatory Rules of Processing Techniques (Sweating and Drying Methods) on Chemical Components of EUB

The correlation between differences in chemical components of EUB and sweating/drying methods is jointly clarified by changes in fingerprints and contents of characteristic components [23]. In terms of fingerprint similarity, the similarity of samples with different drying methods after sweating (S1–S4) is significantly lower than that of fresh-dried samples (S5–S8). Among them, the similarity between S1 (FD4) and S2 (FD) is the lowest (0.715), indicating that differences in drying methods after sweating have a more significant overall impact on components. This difference may be related to biochemical reactions during sweating (e.g., enzymolysis) and drying conditions (temperature, humidity): glycosidases released by cell rupture during sweating may catalyze the transformation of iridoid components or induce chemical reactions such as oxidation and polymerization [24,25], while the low-temperature environment of freeze-drying may retain more enzyme-sensitive components (e.g., PDG), leading to differences in components compared with samples dried at 40 °C [7,8].

In terms of characteristic components, the contents of AU, GPA, CA, and PDG in S7 (XS) are the highest, while the contents of these components in samples after sweating are generally reduced, which is consistent with the conclusions from [6] that “sweating reduces aucubin content” and that from [26] that sweating reduces chlorogenic acid content, suggesting that sweating may promote the degradation or transformation of such components. Under the same drying method, sweating has specific effects on components: in samples dried at 40 °C, the contents of AU and GPA in S1 (FD4) after sweating increase, while those of GP and PDG decrease, which is consistent with previous studies. It is speculated that with the increase of drying temperature, iridoids, on the one hand, reduce glycoside hydrolysis due to the rapid vaporization and loss of water in medicinal materials; on the other hand, glycoside-decomposing enzymes are partially or completely inactivated at appropriate temperatures, reducing the decomposition of glycosides by decomposing enzymes [27,28]. In shade-dried samples, the PDG content in S4 (FY) after sweating is higher than that in S8 (XY) without sweating, suggesting that shade drying may reduce the loss of lignan components during sweating, which is consistent with the previous result that low temperature retains enzyme-sensitive components. These results provide a basis for targeted regulation of EUB components: sun-drying of fresh samples is better for enriching CA and PDG; if the target is iridoid substances, drying at 40 °C after sweating is more appropriate.

### 3.2. Spectrum–Effect Relationship and Mechanism Between Antioxidant Activity and Characteristic Components

The antioxidant activity of EUB is regulated by processing techniques and closely related to the synergistic effects of specific active components. In this study, S4 (FY) exhibited the strongest activity in three free radical scavenging systems (DPPH, ABTS·^+^, and pyrogallol), yet it did not show the highest content of key components—S7 (XS) had higher contents of chlorogenic acid (CA) and pinoresinol diglucoside (PDG), while S5 (XG4) had a higher content of liriodendrin (PD). This “activity–content inconsistency” highlights the complexity of EUB’s antioxidant mechanism, which can be further explained by integrating spectrum–effect relationships and processing data.

First, the balanced synergy of key quantified components in S4 (FY) is the core of its activity advantage. Grey relational analysis revealed specific correlations between different free radical scavenging systems and particular components [29]: DPPH scavenging activity strongly correlated with CA and PDG; ABTS·+ scavenging correlated strongly with CA, PDG, and PD; and pyrogallol scavenging showed a close correlation with PD. In S4 (FY), these components, though not the highest in content, had a coordinated ratio (CA: 3.7036 mg/g, PDG: 4.9187 mg/g, PD: 0.9979 mg/g), forming a “functional complementary network”: CA directly scavenges free radicals via phenolic hydroxyl groups [30] and can enhance antioxidant stability through interaction with biomacromolecules [31]; PDG upregulates the activity of antioxidant enzymes (e.g., SOD, GSH-Px) through the Nrf2/HO-1 pathway and inhibits the NF-κB pathway to reduce inflammatory factor release [32]; geniposidic acid (GPA), with a relatively high content in S4 (FY), mitigates cellular oxidative stress by activating the AKT/Nrf2 signaling pathway [33]; PD enhances non-enzymatic antioxidant defense via the hydrogen atom transfer (HAT) mechanism [34]. This multi-component synergistic effect likely surpasses the scenario in S7 (XS) or S5 (XG4) where individual components are highly abundant but imbalanced, thereby improving overall activity.

Second, the sweating–shade drying process enhances activity through component transformation. The sweating process can activate endogenous glycosidases in EUB [23,24], promoting the conversion of inactive precursors to active forms. For example, hydrolysis of PD’s glycosidic bonds may increase its bioavailability, consistent with the higher PD content in S4 (FY) compared to S8 (XY). Meanwhile, shade drying avoids thermal degradation and photo-oxidation of heat-sensitive components like CA [30], and CA’s binding capacity with molecules can further enhance thermal stability [31]—an advantage over sun-dried S7 (XS), where CA may undergo partial degradation due to light exposure.

Third, unquantified minor components may contribute significantly to activity. HPLC fingerprinting showed multiple common peaks in EUB samples with significant differences in peak areas across processing methods. These unquantified components may include phenolic acid derivatives (e.g., feruloylquinic acid) or lignan oligomers, which are known to possess antioxidant activity [22,35] and can synergize with CA, PDG, and PD. Metabolomic studies on EUB’s sweating process further confirm the presence of such minor components, which may amplify antioxidant effects through additive interactions.

Additionally, S7 (XS), despite having the highest total component content, did not exhibit the strongest antioxidant activity, confirming that antioxidant effects do not depend on total component amount but on the synergism of specific active components [18]. In summary, the strong antioxidant activity of S4 (FY) results from the balanced synergy of key components, processing-induced activation of active forms, and contributions from unquantified phytochemicals. This highlights the holistic nature of EUB’s antioxidant efficacy—coregulated by component contents and processing-mediated interactions rather than dominated by a single high-content component.

### 3.3. Material Basis and Pathway Verification of Hepatoprotective Effect Based on Network Pharmacology

Results of network pharmacology and molecular docking revealed the potential material basis and mechanism of EUB in anti-acute liver injury (ALI). Screening of core targets showed that TNF, IL6, GAPDH, etc., are key intersection targets between EUB components and ALI, and these targets are involved in the regulation of inflammatory responses and oxidative stress. PTGS2 is a key pro-inflammatory factor, and its overexpression can aggravate inflammatory damage in liver tissue [36]; GAPDH participates in cellular energy metabolism, and its dysfunction is closely related to hepatocyte apoptosis [37].

Molecular docking verified the strong binding ability of components such as chlorogenic acid and pinoresinol diglucoside to core targets: Chlorogenic acid showed low binding energies with GAPDH (−9.3 kcal/mol) and PTGS2 (−9.2 kcal/mol), suggesting that it may reduce the production of inflammatory mediators by inhibiting PTGS2 (cyclooxygenase) and protect hepatocytes by stabilizing GAPDH function. The high affinity of pinoresinol diglucoside with GAPDH (−9.9 kcal/mol) and PTGS2 (−10.5 kcal/mol) further supports the role of lignan components in hepatoprotection.

KEGG pathway analysis showed that the AGE–RAGE signaling pathway is the main enriched pathway; its activation can promote ROS production and inflammatory response [38]. EUB components may alleviate liver injury by inhibiting this pathway, which is consistent with the previous conclusion that “oxidative stress is a key link in ALI” [11].

Combined with component content data, S7 (XS) had the highest contents of chlorogenic acid and PDG, while S5 (XG4) showed the highest content of geniposide. We speculate that these samples may exert stronger hepatoprotective effects through the aforementioned component-target-pathway, providing directions for subsequent in vivo experiments.

### 3.4. Limitations and Future Perspectives

The current identification of key targets and regulatory mechanisms of EUB components primarily relies on network pharmacology and molecular docking techniques. These methods are limited by database completeness and computational prediction accuracy, making it difficult to capture low-abundance, transient, or context-specific protein targets. To address these limitations, future studies could integrate emerging technologies to deepen understanding of the material basis and mechanism of action of EUB. For instance, PROTAC (Proteolysis-Targeting Chimera) probe technology, as a powerful tool for targeted protein degradation and identification, can specifically capture and characterize direct protein targets of key EUB components (e.g., chlorogenic acid, pinoresinol diglucoside) in physiological environments. This technology enables the discovery of previously unrecognized target proteins, particularly those with weak or transient interactions that are easily missed by traditional molecular docking or affinity purification methods [39,40].

Furthermore, integrating proteomics technology will facilitate the systematic analysis of global protein expression profiles and post-translational modifications of EUB under different processing conditions (e.g., sweating–drying) or protein changes in acute liver injury (ALI) models treated with EUB components [41]. This approach can not only identify key enzymes involved in component transformation during processing (e.g., glycosidases or oxidases) but also reveal downstream signaling molecules and regulatory networks mediated by active components of EUB, thereby bridging the gap between component changes and pharmacological effects. By combining PROTAC probe technology with proteomics, we can achieve more accurate and comprehensive identification of key targets and their dynamic interactions in EUB, providing a more solid scientific basis for clarifying processing–efficacy relationships and guiding the clinical application of EUB.

## 4. Materials and Methods

### 4.1. Plant Materials and Reagents

Fresh *Eucommia ulmoides* (EUB) Oliv. bark samples were collected in Zhijin, Guizhou, China (N 26°40′12.0″ E 105°46′48.0″). Professor Wei Li (Guizhou University of Traditional Chinese Medicine) identified them as the species from the family *Eucommiaceae*.

The solvents used in this study included absolute ethanol (Kemao Chemical Reagent Co., Ltd., Tianjin, China; Lot. 20241128), phosphoric acid (Kermel Chemical Reagent Co., Ltd., Tianjin, China; Lot. 20240111), and acetonitrile (Kermel Chemical Reagent Co., Ltd., Tianjin, China; Lot. 20240225).

The reference standards included aucubin (AU, Lot. MUST-23092514), geniposidic acid (GPA, Lot. MUST-24010211), geniposide (GP, Lot. MUST-24050411), chlorogenic acid (CA, Lot. MUST-24013002), pinoresinol diglucoside (PDG, Lot. MUST-24030121), and liriodendrin (PD, Lot. MUST-24080903), all purchased from Chengdu Mansite Biotechnology Co., Ltd. (Chengdu, China).

The chemical reagents used in this study included 2,2-diphenyl-1-picrylhydrazyl (DPPH, Shanghai Yuanye Bio-Technology Co., Ltd., Shanghai, China; Lot. N04HS200060), 2,2′-azino-bis (3-ethylbenzothiazoline-6-sulfonic acid) (ABTS·+, Shanghai Yuanye Bio-Technology Co., Ltd., Shanghai, China; Lot. N04HS199908), pyrogallol (Aladdin Biochemical Technology Co., Ltd., Shanghai, China; Lot. J2111111), Tris-HCl buffer (Feijing Biotechnology Co., Ltd., Fuzhou, China; Lot. 20241029), and potassium persulfate (McLean Chemical Reagent Co., Ltd., Shanghai, China; Lot. C16635346).

All chemicals and solvents were of analytical grade or HPLC grade.

### 4.2. Sample Preparation

**Figure 11:**
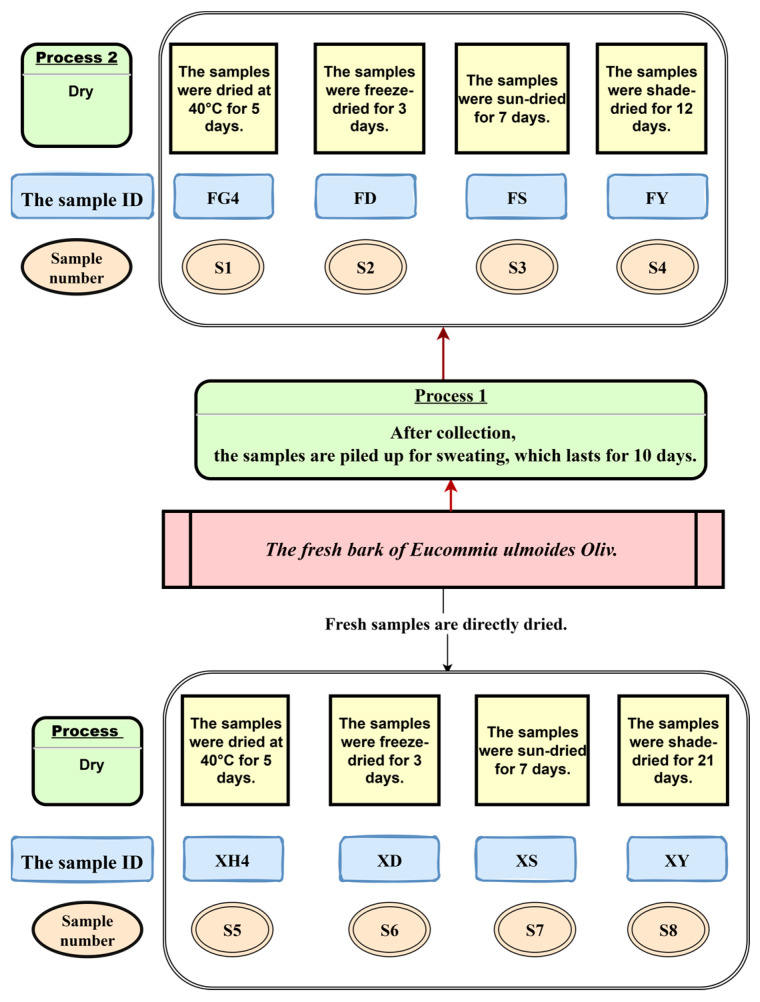
The processing of EUB samples.

The processing of EUB samples was conducted according to the protocol developed by our research team, with the detailed procedure shown in Figure 11. After processing, the samples were crushed using a micropulverizer, sieved through a 50-mesh sieve, and the resulting powder was collected.

#### 4.2.1. Sample Preparation for HPLC Analysis

For HPLC fingerprinting: 0.5 g of the sample + 10 mL of 50% ethanol was extracted using ultrasonic extraction (60 min) with an ultrasonic cleaner (SK8200LHC, Keda Shanghai, Germany) and filtered through a 0.22 μm membrane.

For component quantification: 0.5 g of sample + 25 mL of 70% ethanol using the same extraction procedure as described above.

Reference standards were prepared as described above.

#### 4.2.2. Preparation of Antioxidant Samples

- DPPH radical scavenging test sample: With 0.25 g of sample + 25 mL of 50% ethanol, perform ultrasonic treatment (53 KHz, 40%, 30 min), and reweigh and replenish to the original weight to obtain a 10 mg/mL EUB extract. Take an appropriate amount of the extract and dilute with 50% ethanol to prepare test sample solutions with concentrations of 5, 2.5, 1.25, 0.625, and 0.3125 mg/mL respectively.
- ABTS·+ radical scavenging test sample: 0.040 g of sample + 40 mL of 50% ethanol wasa extracted using the same method as described above to obtain a 1 mg/mL EUB extract. Dilute similarly to concentrations of 0.5, 0.4, 0.3, 0.2, and 0.1 mg/mL.
- Pyrogallol scavenging test sample: 4 g of sample + 25 mL of 50% ethanol was extracted using the same method as described above to obtain a 160 mg/mL EUB extract. Dilute similarly to concentrations of 50, 40, 30, 20, and 10 mg/mL.

### 4.3. Moisture Determination

Moisture was determined using the “oven-drying method” according to General Chapter 0832 of Volume IV of the *Chinese Pharmacopoeia (2020 Edition).*

Precisely weigh 2 g of the test sample and place it in a flat weighing bottle dried to constant weight (thickness ≤ 5 mm). Dry at 100–105 °C for 5 h with the lid open, then close the lid, transfer to a desiccator, allow to cool for 30 min, and weigh precisely. Dry again at the above temperature for 1 h, cool, and weigh, repeating until the difference between two consecutive weighings does not exceed 5 mg. The moisture content (%) of the test sample was calculated based on the weight loss.

According to the *Chinese Pharmacopoeia*, the moisture content of this product shall not exceed 13%.

### 4.4. HPLC Analysis

#### 4.4.1. Establishment of EUB Fingerprint

The HPLC fingerprint was established using a SHIMADZU LC-16 high-performance liquid chromatography-diode array detection (HPLC-DAD) system, equipped with an Agilent ZORBAX SB-C18 column (250 mm × 4.6 mm × 5 μm). Mobile phase A was 0.1% phosphoric acid aqueous solution, and mobile phase B was acetonitrile. The gradient elution program was as follows: 5–15% B at 0–40 min; 15–35% B at 40–70 min; 35–5% B at 70–75 min. The flow rate was 1 mL/min, the column temperature was maintained at 30 °C, the detection wavelength was 220 nm, and the injection volume was 10 μL.

#### 4.4.2. Methodological Validation of Fingerprint

This section is based on the preliminary research of our research team, with relevant details provided in [42].

#### 4.4.3. Determination of Sample Contents

The contents of AU, GPA, GP, CA, PDG, and PD in samples were detected by HPLC. The detection instrument was a SHIMADZU LC-16 HPLC-DAD system, equipped with a Hydrosphere C18 column. Chromatographic conditions were the same as those described in Section 4.4.1.

Selection criteria for quantified components are as follows:

- They are characteristic markers of EUB, andAU, GPA, and GP as representative iridoid glycosides; among lignans, PDG is a major component specific to this species, and PD is a major bioactive lignan in EUB;
- They have well-documented antioxidant and hepatoprotective activities, which are directly related to the study’s focus on anti-ALI and antioxidant mechanisms;
- Their stable chromatographic peaks in preliminary HPLC fingerprint analysis (Section 2.2) ensured reliable quantification;
- They were identified as high-degree active components in network pharmacology analysis, showing strong associations with ALI targets and pathways (Section 2.6).

#### 4.4.4. Methodological Validation of Content Determination

The methodological validation was conducted as described in Section 4.4.2.

### 4.5. Investigation of Antioxidant Activity

#### 4.5.1. Preparation of Reference Solution

Weigh 0.050 g of vitamin C (ascorbic acid) reference standard, place in 250 mL volumetric flask, make up to the volume with distilled water to prepare vitamin C solution at 200 μg/mL concentration, then dilute with distilled water, as shown in Table 8. 

#### 4.5.2. Preparation of Working Solutions

DPPH working solution: Take 4.0 mg of DPPH, dissolve it in absolute ethanol, dilute to volume in 100 mL volumetric flask, and ultrasonically dissolve (53 KHz, 40%, 30–60 s) to prepare 0.04 mg/mL solution. Store in the dark.

ABTS·+ working solution: Take 0.096 g of ABTS·+, dissolve it in distilled water, dilute to volume in 25 mL volumetric flask, and ultrasonically dissolve under the same conditions to prepare a 7 mmol/L ABTS·+ radical solution. Take 0.033 g of K_2_S_2_O_8_, dissolve it in distilled water, dilute to volume in 23 mL volumetric flask to prepare 4.9 mmol/L K_2_S_2_O_8_ solution. Mix the two solutions in equal proportions to serve as the stock solution, place it in the dark for 16 h, then dilute with absolute ethanol to adjust the absorbance at 734 nm to 0.70 ± 0.02. This is the ABTS·+ working solution; store in the dark.

Pyrogallol working solution: Take 0.065 g pyrogallol, dissolve it in absolute ethanol, dilute to volume in 50 mL volumetric flask, and ultrasonicate for 30 s to obtain 1.3 mg/mL solution. Store in the dark.

#### 4.5.3. Determination of A_0_

Distilled water was used as the blank control for all measurements. The absorbance (A_0_) of DPPH was measured at 517 nm; ABTS was measured at a wavelength of 734 nm; and pyrogallol was measured at 325 nm.

#### 4.5.4. Determination of A

- Precisely pipette 2.0 mL of each test sample solution and 2.0 mL of DPPH working solution and mix thoroughly by shaking. After vortexing, incubate at room temperature in the dark for 30 min, then measure the absorbance (A) at 517 nm.
- Accurately draw 2.0 mL of each test sample solution and 2.0 mL of ABTS·+ working solution and mix well. Allow to react for 6 min at room temperature protected from light, then determine the absorbance (A) at 734 nm.
- Precisely measure 25 mL of Tris-HCl and dilute to 500 mL with distilled water in a volumetric flask to prepare the buffer solution. Combine 1.0 mL of sample solution with 4.5 mL of buffer in a test tube; measure 0.4 mL of pyrogallol into a small beaker. Place both the flask and test tube in a 25 °C water bath, protect from light for 5 min, then mix the two solutions and measure the absorbance (A) at 325 nm.

#### 4.5.5. Determination of A_x_

For both DPPH and ABTS·+ assays: Mix 2.0 mL of absolute ethanol with 2.0 mL of test sample solution and shake thoroughly. After vortex-mixing, incubate at room temperature in the dark for 30 min, then measure absorbance (A_x_) at the corresponding wavelength as described above. For the pyrogallol assay: Combine 4.5 mL of the prepared buffer, 1 mL of test sample solution, and 0.4 mL of the pyrogallol working solution, then determine absorbance at the corresponding wavelength.

### 4.6. Calculation of Free Radical Scavenging Rate

The calculation formula of free radical scavenging rate is as follows:

Free Radical Scavenging Rate (%) = [1 − (A − A_x_)]/A_0_ × 100%

Note: A = absorbance of test sample; A_x_ = absorbance of blank group; A_0_ = absorbance of control group.

### 4.7. Network Pharmacology of Acute Liver Injury

#### 4.7.1. Acquisition of Components and Disease Targets

Thirty-three active components of EUB, including chlorogenic acid, geniposidic acid, pinoresinol diglucoside, and aucubin (Table 9), were identified via literature retrieval. Targets were screened and validated using the UniProt database (https://www.uniprot.org, accessed on 16 November 2024) and Swiss Target Prediction database (http://www.swisstargetprediction.ch, accessed on 16 November 2024). Acute liver injury-related target genes were retrieved from the OMIM database (https://www.omim.org, accessed on 16 November 2024) and GeneCards database (https://auth.lifemapsc.com, accessed on 16 November 2024) with the keyword “Acute liver injury”.

The results of the two screenings were integrated and duplicates were eliminated to obtain the core target gene set for acute liver injury. The intersection of drug active component targets and disease targets was subjected to visual analysis.

#### 4.7.2. Construction and Core Screening of Protein–Protein Interaction (PPI) Network

The intersection targets of drugs and diseases were imported into the STRING database (https://cn.string-db.org, accessed on 18 November 2024), with the species set to Homo sapiens. Disconnected gene targets were hidden, and the confidence score was set to >0.4 to construct the Protein–Protein Interaction (PPI) network. Cytoscape software (Version 3.10.1) was used to map the active component-target network of EUB. The “Network.txt” and “Tape.txt” files were created and imported into Cytoscape to analyze the relationships between active components of EUB and their respective targets. Core targets and active components were screened based on degree values.

#### 4.7.3. Pathway Analysis

GO and KEGG analyses were performed on the intersection genes of drugs and diseases using the DAVID database (https://davidbioinformatics.nih.gov/, accessed on 18 November 2024). Bar plots of the enrichment results were generated with SRplot (https://www.bioinformatics.com.cn/, accessed on 18 November 2024).

### 4.8. Molecular Docking

Molecular docking verification was performed based on the core targets and active components screened in Section 4.7.2;. Appropriate target proteins were selected and exported from the UniProt website following the criteria of minimum “RESOLUTION”, minimum “CHAIN” count, and longest chain length. The 3D structure files of active components were downloaded from the PubChem database (https://pubchem.ncbi.nlm.nih.gov/, accessed on 21 November 2024), and the PDB formats of core targets and active components were exported using PyMol software (Version 3.1.0) for subsequent use. Finally, molecular docking was conducted via Autodock Vina in AMDock software (Version 1.0), and the results were visualized using PyMol.

### 4.9. Data Processing

Similarity analysis of fingerprint profiles was performed using the Traditional Chinese Medicine Chromatographic Fingerprint Similarity Evaluation System (Version 2004A). Content determination data were processed with WPS Excel software (Version 12.1.0), and nonlinear fitting of sample antioxidant activity values was conducted using GraphPad Prism (Version 9.5.0). The sample IC_50_ was calculated via the “log(inhibitor) vs. normalized response–variable slope” model. Content determination results and IC_50_ values were imported into SPSSPro (https://www.spsspro.com/, accessed on 13 May 2025) for online data analysis. Taking the antioxidant index IC_50_ as the mother sequence and each component’s determination value as characteristic sequences, dimensionless processing of raw data was performed using the mean normalization method. With *P* set to 0.5, the correlation degree “**r**” was calculated, and data were visualized using SRplot (https://www.bioinformatics.com.cn/, accessed on 13 May 2025) after collation.

## 5. Conclusions

This study, via spectrum–effect relationship combined with multiple technical approaches, clarifies that sweating and drying processes significantly regulate the chemical components and pharmacodynamic activities of *Eucommia ulmoides* Oliv. (EUB). Specifically, drying methods exert a notable impact on the components of EUB after sweating (e.g., the similarity of HPLC fingerprints between 40 °C-dried and freeze-dried samples is only 0.715). Sun-drying of samples is more conducive to the accumulation of key antioxidant and hepatoprotective components such as chlorogenic acid (CA) and pinoresinol diglucoside (PDG). Samples dried in the shade after sweating exhibit the strongest scavenging capacities against DPPH, ABTS·+, and pyrogallol free radicals, and their activity is closely related to the synergistic effects of components including CA, PDG, GPA, and PD.

Network pharmacology and molecular docking verify that EUB exerts anti-acute liver injury effects through active components such as CA and PDG, which act on core targets including TNF, IL6, PTGS2, and GAPDH, and regulate pathways like AGE–RAGE.

In conclusion, this study provides a scientific basis for the standardization of “sweat–g-drying” processes of EUB and its precise application in anti-acute liver injury.

## Figures and Tables

**Table 1:** Moisture determination results of EUB under different processing methods.

Number	Sample ID	Sample Preparation Process	Moisture Content (%)
S1	FG4	The samples were dried at 40 °C for 5 days after 10 days of sweating.	4.44
S2	FD	The samples were freeze-dried for 3 days after 10 days of sweating.	4.21
S3	FS	The samples were sun-dried for 7 days after 10 days of sweating.	5.30
S4	FY	The samples were shade-dried for 12 days after 10 days of sweating.	5.17
S5	XH4	The samples were dried at 40 °C for 5 days.	4.45
S6	XD	The samples were freeze-dried for 3 days.	6.10
S7	XS	The samples were sun-dried for 7 days.	7.44
S8	XY	The samples were shade-dried for 12 days.	6.12

**Table 2:** Content determination results of six components in EUB under different geographical origin processing methods. (mg/g, n=3). Note: n=3 represents technical replicates; data are ex-pressed as mean ± standard deviation, used to reflect HPLC quantification precision and component variation trends).

Samples	AU	GPA	GP	CA	PDG	PD
S1	23.9871 ± 0.0366	18.2294 ± 0.0326	1.3218 ± 0.0183	3.2374 ± 0.0175	4.1987 ± 0.0150	0.5041 ± 0.0006
S2	9.7823 ± 0.0430	6.1853 ± 0.0289	1.5053 ± 0.0187	2.2208 ± 0.0072	5.3419 ± 0.0160	1.0918 ± 0.0069
S3	21.8668 ± 0.5201	3.3948 ± 0.0484	0.4075 ± 0.0101	2.5850 ± 0.0684	3.2480 ± 0.0155	0.7818 ± 0.0065
S4	11.7792 ± 0.0226	10.8488 ± 0.0214	1.4070 ± 0.0187	3.7036 ± 0.0162	4.9187 ± 0.0181	0.9979 ± 0.0004
S5	8.4761 ± 0.2314	7.6489 ± 0.1063	6.8651 ± 0.0138	3.9901 ± 0.1179	5.7927 ± 0.0498	1.1125 ± 0.0034
S6	16.6944 ± 0.0870	16.9340 ± 0.0660	4.4749 ± 0.0392	4.3495 ± 0.0335	6.8109 ± 0.0168	1.0953 ± 0.0018
S7	32.1872 ± 0.0960	20.9983 ± 0.0762	0.4396 ± 0.0094	4.3547 ± 0.0700	6.9276 ± 0.0041	0.6088 ± 0.0020
S8	11.6269 ± 0.4148	14.7512 ± 0.1440	0.6862 ± 0.0145	3.8766 ± 0.0483	3.9811 ± 0.0256	0.4930 ± 0.0030

**Table 3:** Antioxidant determination results of EUB samples (n = 3, Note: IC_50_ was calculated by fitting the “log(inhibitor) vs. normalized response-variable slope” model; the goodness of fit is shown in parentheses, used to reflect activity trends).

Samples ID	IC_50_(mg/mL)		
	DPPH	ABTS·+	Pyrogallol
VC	0.024	0.002	0.064
S1	1.108	0.241	14.870
S2	3.381	0.210	35.840
S3	0.973	0.140	21.420
S4	0.828	0.134	14.200
S5	1.607	0.174	14.220
S6	2.073	0.151	51.120
S7	2.042	0.139	16.360
S8	2.267	0.160	16.200

**Table 4:** Degradation value of components in EUB.

Number	Degree Value	Constituents	Structure Type
D33	55	Geniposide	Iridoids
D32	50	Caffeic acid	Phenylpropanoids
D21	29	quercetin	Flavonoids
D31	25	Chlorogenic acid	Phenylpropanoids
D26	22	rutin	Flavonoids
D4	22	kaempferol	Flavonoids
D3	20	β-sitosterol	Steroids
D24	16	Syringetin	Flavonoids
D12	12	(−)-Tabernemontanine	Alkaloids
D17	8	(+)-Eudesmin	Lignans
D18	7	Dehydrodiconiferyl alcohol	Lignans
D1	7	40957-99-1	Lignans
D15	6	Quinidine	Alkaloids
D25	6	Aucubin	Iridoids
D27	6	(+)-Pinoresinol-Di-O-β-D-Glucoside	Lignans
D29	6	protocatechuic acid	Phenolic acids
D6	6	Erythraline	Alkaloids
D7	5	AIDS214634	Lignins
D19	5	hirsutin	Flavonoids
D11	5	(+)-medioresinol	Lignans
D30	4	Eucommiol	Alicyclic hydrocarbons
D8	3	3-β-Hydroxymethyllenetanshiquinone	Quinones
D5	3	olivil	Lignans
D22	3	β-carotene	Tetraterpenes
D14	3	Dehydrodiconiferyl alcohol 4, **γ**-di-O-β-D-glucopyanoside	Lignans
D13	3	Cyclopamine	Alkaloids
D10	3	Yangambin	Lignans
D9	2	ent-Epicatechin	Flavonoids
D28	2	Geniposide	Iridoids
D23	2	(E)-3-[4-[(1R,2R)-2-hydroxy-2-(4-hydroxy-3-methoxy-phenyl)-1-methylol-ethoxy]-3-methoxy-phenyl] acrolein	Lignans
D20	2	liriodendrin	Lignans
D2	2	Mairin	Triterpenoids
D16	2	Helenalin	Sesquiterpenes

**Table 5:** The CytoNCA plugin screens out the top 10 core target proteins through two median filters.

Target Proteins	Degree	Betweenness	Closeness
TNF	206.0	2263.5625	0.65686274
IL6	204.0	2183.5693	0.6547231
GAPDH	204.0	3988.2634	0.6611842
IL1B	178.0	1489.3212	0.62616825
TP53	176.0	1482.7214	0.6036036
PTGS2	170.0	1842.8794	0.6203704
EGFR	166.0	1384.4694	0.6128049
STAT3	158.0	1247.3453	0.6036036
ESR1	156.0	1919.2544	0.60542166
PPARG	148.0	801.0384	0.57758623

**Table 6:** Selection of active constituents from EUB.

Number	Degree Value	Constituents	Structure Type
D33	55	Geniposide	Iridoids
D32	50	Caffeic acid	Phenylpropanoids
D21	29	quercetin	Flavonoids
D31	25	Chlorogenic acid	Phenylpropanoids
D26	22	rutin	Flavonoids
D4	22	kaempferol	Flavonoids
D3	20	β-sitosterol	Steroids
D24	16	Syringetin	Flavonoids
D12	12	(−)-Tabernemontanine	Alkaloids
D17	8	(+)-Eudesmin	Lignans
D18	7	Dehydrodiconiferyl alcohol	Lignans
D1	7	40957-99-1	Lignans
D15	6	Quinidine	Alkaloids
D25	6	Aucubin	Iridoids
D27	6	(+)-Pinoresinol-Di-O-β-D-Glucoside	Lignans
D29	6	protocatechuic acid	Phenolic acids
D6	6	Erythraline	Alkaloids

**Table 7:** Results of Molecular Docking between Components of EUB and Potential Targets of Acute Liver Injury and Active Ingredients.

Constituents	TNF	IL6	GAPDH	IL1B	TP53	PTGS2	EGFR	STAT3	ESR1	PPARG
Geniposide	−6	−6	−8.8	−6.7	−5.63	−7.7	−7.6	−7.2	−6.6	−7.3
Caffeic acid	−6	−6	−6.6	−5.4	−8.48	−6.9	−6.8	−5.7	−6.1	−6.3
quercetin	−7	−6.7	−7.7	−6.3	−7.34	−9.7	−8.6	−8.4	−7.5	−7.3
Chlorogenic acid	−7	−6.6	−9.3	−6.7	−6.24	−9.2	−8.3	−7.4	−7.2	−7.4
rutin	−7.1	−6.8	−10.1	−7.2	−6.13	−9.3	−8.6	−8.8	−7.8	−9
kaempferol	−6.8	−6.4	−7.2	−6.1	−7.02	−8.7	−8.3	−8.2	−6.9	−7.6
β-sitosterol	−6	−6.8	−8.8	−6.7	−8.02	−9.7	−9.2	−7.5	−7.1	−7.9
Syringetin	−6.7	−6.6	−9.3	−6.8	−7.37	−8.5	−8.1	−8.3	−6.9	−8
(−)-Tabernemontanine	−6.5	−6.8	−8.9	−7.1	−8.16	−7.6	−7.7	−7.3	−7.4	−8.4
(+)-Eudesmin	−6	−6.1	−8.5	−6.5	−6.76	−8.4	−8.6	−6.9	−7	−7.2
Dehydrodiconiferyl alcohol	−6.6	−6.8	−8.6	−6.7	−8.48	−9.1	−8	−7.3	−6.3	−7.3
40957-99-1	−6.7	−6.7	−9.5	−6.8	−7.92	−8.4	−8.6	−7.8	−6.8	−8.1
Quinidine	−6.1	−6.5	−9	−6.3	−7.45	−8.8	−9	−6.7	−6.6	−7.4
Aucubin	−6.6	−6.1	−8.8	−6.6	−7.06	−7.8	−7.3	−6.6	−6.4	−7.2
(+)-Pinoresinol-Di-O-β-D-Glucoside	−7.2	−6.8	−9.9	−7.6	−5.99	−10.5	−9.1	−8.8	−8.3	−8.8
protocatechuic acid	−5.7	−5.8	−6	−5	−6.09	−6.8	−6	−5.5	−5.4	−5.9
Erythraline	−6	−7.1	−8.8	−6.8	−7.83	−8.4	−8.1	−7.4	−6.6	−8.2

**Table 8:** Dilution concentrations of VC for different oxidation indices.

VC Dilution Concentrations		
DPPH (μg/mL)	ABTS·+ (μg/mL)	pyrogallol (μg/mL)
50	6	150
25	5	125
12.5	4	100
6.25	3	75
3.125	2	50

**Table 9:** Constituents of EUB.

Number	Constituents	Structure Type
D1	Medioresinol	Lignans
D2	Mairin	Triterpenoids
D3	β-sitosterol	Steroids
D4	kaempferol	Flavonoids
D5	olivil	Lignans
D6	Erythraline	Alkaloids
D7	AIDS214634	Lignins
D8	3-β-Hydroxymethyllenetanshiquinone	Quinones
D9	ent-Epicatechin	Flavonoids
D10	Yangambin	Lignans
D11	(+)-medioresinol	Lignans
D12	(−)-Tabernemontanine	Alkaloids
D13	Cyclopamine	Alkaloids
D14	Dehydrodiconiferyl alcohol 4, **γ**-di-O-β-D-glucopyanoside	Lignans
D15	Quinidine	Alkaloids
D16	Helenalin	Sesquiterpenes
D17	(+)-Eudesmin	Lignans
D18	Dehydrodiconiferyl alcohol	Lignans
D19	hirsutint	Flavonoids
D20	liriodendrin	Lignans
D21	quercetin	Flavonoids
D22	β-carotene	Tetraterpenes
D23	(E)-3-[4-[(1R,2R)-2-hydroxy-2-(4-hydroxy-3-methoxy-phenyl)-1-methylol-ethoxy]-3-methoxy-phenyl] acrolein	Lignans
D24	Syringetin	Flavonoids
D25	Aucubin	Iridoids
D26	rutin	Flavonoids
D27	(+)-Pinoresinol-Di-O-β-D-Glucoside	Lignans
D28	Geniposidic acid	Iridoids
D29	protocatechuic acid	Phenolic acids
D30	Eucommiol	Alicyclic hydrocarbons
D31	Chlorogenic acid	Phenylpropanoids
D32	Caffeic acid	Phenylpropanoids
D33	Geniposide	Iridoids

## Data Availability

The original contributions presented in this study are included in the article. Further inquiries can be directed to the corresponding author.

## Abbreviations

The following abbreviations are used in this manuscript:

EUB	*Eucommia ulmoides*
ALI	Acute liver injury
HPLC	High-Performance Liquid Chromatography
DPPH	2,2-Diphenyl-1-picrylhydrazyl
ABTS	2,2′-Azino-bis (3-ethylbenzothiazoline-6-sulfonic acid)
CA	Chlorogenic acid
PDG	Pinoresinol diglucoside
AU	Aucubin
GPA	Geniposidic acid
GP	Geniposide
PD	Liriodendrin
TNF	Tumor Necrosis Factor
PTGS2	Prostaglandin–Endoperoxide Synthase 2
IL6	Interleukin 6
IL1B	Interleukin 1 β
TP53	Tumor Protein 53
EGFR	Epidermal Growth Factor Receptor
STAT3	Signal Transducer and Activator of Transcription 3
ESR1	Estrogen Receptor 1
PPARG	Peroxisome Proliferator-Activated Receptor Gamma
GAPDH	Glyceraldehyde-3-Phosphate Dehydrogenase
AGE-RAGE	Advanced Glycation End Products–Receptor for Advanced Glycation End Products
APAP	Acetaminophen
ROS	Reactive Oxygen Species
SOD	Superoxide Dismutase
GSH	Glutathione
MDA	Malondialdehyde
ALT	Alanine Transaminase
AST	Aspartate Transaminase
Nrf2	Nuclear Factor Erythroid-2-Related Factor
NF-κB	Nuclear Factor-kappa B
FG4	The samples were dried at 40 °C for 5 days after 10 days of sweating.
FD	The samples were freeze-dried for 3 days after 10 days of sweating.
FS	The samples were sun-dried for 7 days after 10 days of sweating.
FY	The samples were shade-dried for 12 days after 10 days of sweating.
XG4	The samples were dried at 40 °C for 5 days.
XD	The samples were freeze-dried for 3 days.
XS	The samples were sun-dried for 7 days.
XY	The samples were shade-dried for 12 days.
VC	Vitamin C
IC_50_	Half maximal Inhibitory Concentration
PPI	Protein–Protein Interaction
GO	Gene Ontology
KEGG	Kyoto Encyclopedia of Genes and Genomes
HAT	Hydrogen atom transfer
ET	Electron transfer
ASM	Sadenosylmethionine
PI3K–AKT	Phosphatidylinositol 3-kinase–Protein Kinase B
